# Incidence and risk factors for retinopathy of prematurity in premature, extremely low birth weight and extremely low gestational age infants

**DOI:** 10.1186/s12886-022-02591-9

**Published:** 2022-09-13

**Authors:** Ozlem Eski Yucel, Bilge Eraydin, Leyla Niyaz, Ozlem Terzi

**Affiliations:** 1 Department of Ophthalmology, Ondokuz Mayis University Faculty of Medicine, 55139, Atakum, Samsun, Turkey; 2 Department of Ophthalmology, Bafra State Hospital, Samsun, Turkey; 3 Department of Public Health, Ondokuz Mayis University Faculty of Medicine, Samsun, Turkey

**Keywords:** Extremely low gestational age infants, Extremely low birth weight infants, Premature, Retinopathy of prematurity, ROP

## Abstract

**Background:**

The aim of the study was to determine the incidence and risk factors of retinopathy of prematurity (ROP) in premature, extremely low birth weight (BW, ELBW) and extremely low gestational age (GA, ELGA) infants.

**Methods:**

The medical records of preterm infants who were screened for ROP between January 2012 and December 2020 were retrospectively reviewed. Only one eye of each infant with higher grade ROP was included in the study. BW; GA; medical characteristics; the presence, severity, and need for treatment of ROP were recorded. Infants were divided into groups according to BW (≤1000 g, 1001-1750 g, > 1750 g) and GA (≤25w, 26-28w, 29-31w, 32-34w, ≥35w) and data were analyzed.

**Results:**

Data of 2186 infants were evaluated. The overall incidences of any stage ROP and ROP requiring treatment were 43.5 and 8.0%, respectively. These rates were 81.1 and 23.9% in ELBW (≤1000 g) infants and were 92.9 and 64.3% in ELGA (≤25w) infants, respectively. The rates of ROP, the median duration of oxygen therapy and systemic diseases increased significantly as BW and GA decreased. The median duration of oxygen therapy and the rates of sepsis, pulmonary dysplasia (BPD), and intraventricular hemorrhage (IVH) were statistically higher in infants with ROP compared to those without ROP (*p* < 0.001). Multivariate regression analysis demonstrated that low BW and GA; prolonged duration of oxygen therapy; presence of PDA and necrotizing enterocolitis (NEC) were important risk factors for ROP.

**Conclusions:**

ELBW and ELGA infants develop higher rates of ROP and severe ROP. Prolonged duration of oxygen therapy, the presence of concomitant neonatal sepsis, BPD, IVH, PDA, and NEC further increases the risk of ROP.

## Introduction

The survival rates of very premature and low birth weight (BW) infants who are at risk of mortality and morbidity, including retinopathy of prematurity (ROP), have increased with advances in neonatal intensive care units [1, 2]. ROP is a leading cause of childhood blindness [3]. The incidence of ROP varies from country to country [4–9]. Several risk factors such as low BW [4–6, 8–10], low gestational age (GA) [4–6, 8–10], high oxygen saturation [11], mechanical ventilation [5, 6], phototherapy [8], intraventricular hemorrhage (IVH) [6, 7], anemia [6], blood transfusion [4, 6, 8, 9], patent ductus arteriosus (PDA) [6, 7, 9], respiratory distress syndrome (RDS) [6, 9], broncho pulmonary dysplasia (BPD) [6], necrotizing enterocolitis (NEC) [5, 6, 9], sepsis [4, 9], low Apgar scores [6] have been associated with the development of ROP.

Some studies have been published to determine the incidence and risk factors of ROP in extremely low BW (ELBW) infants [10, 12–14]. We planned this study to determine the incidence and risk factors of ROP in premature, ELBW and extremely low GA (ELGA) infants followed in our hospital, which is a tertiary referral and treatment center.

## Materials and methods

### Study design

This retrospective study was conducted at the Ondokuz Mayis University Hospital. The medical records of preterm infants who were screened for ROP between January 2012 and December 2020 were reviewed.

### Subjects

Premature infants with a BW of ≤1700 g (g) and a GA of < 34 weeks (w), and with a BW of > 1700 g or a GA of ≥34 w who have received cardiopulmonary support therapy or were considered at risk for the development of ROP by the neonatologists were included in the study [15]. Premature infants who did not complete all screening sessions and had missing data were excluded from the study. This study was approved by the Ondokuz Mayis University Clinical Research Ethics Committee and was performed according to the principles of Declaration of Helsinki. Informed consent was obtained from the parents before the ophthalmological examination for ROP.

### Data collection

ROP examinations were performed by two ophthalmologists (OEY and LN) experienced in ROP. BW, GA, multiple gestations, medical histories, the presence and severity of ROP, the need and type of treatment for ROP were recorded. ROP examination included assessment of pupillary dilation; vitreous clarity; presence of plus disease; presence, stage, location, and extent of ROP disease. ROP was classified according to the international classification of retinopathy [16]. ROP type 1 was defined as any stage ROP with plus disease (a degree of dilation and tortuosity of the posterior retinal blood vessels) at zone I, stage 3 ROP without plus disease at zone I, and stage 2 or 3 ROP with plus disease at zone II. ROP type 2 was defined as stage 1 or 2 ROP without plus disease at zone I and stage 3 ROP without plus disease at zone II [17]. ROP cases except types 1 and 2 were defined as mild ROP. Premature infants divided into groups according to BW (≤1000 g, 1001–1750 g, > 1750 g) and GA (≤25 w, 26–28 w, 29–31 w, 32–34 w, ≥35 w) and data were analyzed. Infants with a BW of ≤1000 g were considered as ELBW infants, and infants with a GA of ≤25 w were considered as ELGA infants [18, 19]. Data from only one eye of each infant with higher grade ROP were evaluated.

### Statistical analysis

Statistical analysis was performed using V22 IBM SPSS (SPSS Inc., Chicago, IL, USA). The compliance of the data to normal distribution was evaluated with the Kolmogorov Smirnov test. Non-parametric tests were used because the data are not normally distributed. The Kruskal-Wallis and Mann-Whitney U tests were used for continuous independent variables and Chi-square test used for categorical variables. ​​ The results were given as median (minimum-maximum) and frequency (%). Binary logistic regression analysis was performed to determine the factors associated with ROP development. *P* values of < 0.05 were considered to be statistically significant.

## Results

### General characteristics of the patients

Two thousand one hundred and eighty-six premature infants with a median GA of 31 (22–38) w and a mean BW of 1500 (540–3450) g were included in the study. There were 347 ELBW infants with a median BW of 800 (540–1000) g and 28 ELGA infants with a median GA of 24 (22–25) w. Of the cases, 1075 (49.2%) were girls and 1111 (50.8%) were boys; 1571 (71.9%) were single, 529 (24.2%) were twins, and 86 (3.9%) were triplets. The distributions of BW and GA, and characteristics of infants in the groups are given in Table 1.

**Table 1:** Demographic characteristics and systemic diseases of infants according to birth weight and gestational age

	n	Gender Male / Female n (%)	Multiple gestations n (%)	BW (g) med (min-max)	GA (w) med (min-max)	Duration of O2 therapy (d), med (min-max)	Sepsis n (%)	BPD n (%)	IVH (≥Grade II) n (%)	NEC n (%)	PDA n (%)
BW (g)											
≤1000	347	147 (42.4) / 200 (57.6)	83 (23.9)	800 (540-1000)	28 (22-34)	47 (4-250)	66 (19)	97 (28)	40 (11.5)	22 (6.3)	78 (22.5)
1001-1750	1274	651 (51.1) / 623 (48.9)	359 (28.2)	1460 (1002-1750)	31 (26-38)	29 (2-180)	128 (10)	166 (13)	85 (6.7)	34 (2.7)	222 (17.4)
>1750	565	313 (55.4) / 252 (44.6)	173 (30.6)	2000 (1760-3450)	33 (26-38)	15 (1-290)	15 (2.7)	50(8.8)	28 (4.9)	13 (2.3)	61 (10.8)
p		0.001^a^	0.092	<0.001^a^	<0.001^a^	<0.001^a^	<0.001^a^	<0.001^a^	0.027^a^	0.028^a^	0.027^a^
GA (w)											
≤25	28	15 (53.6) / 13 (46.4)	9 (32.1)	740 (550-1010)	24 (22-25)	61 (23-90)	11 (39.3)	13 (46.4)	13 (46.4)	2 (7.1)	12 (42.9)
26-28	344	166 (48.3) / 178 (51.7)	71 (20.6)	950 (540-1540)	28 (26-28)	46 (2-250)	52 (15.1)	84 (24.4)	43 (12.5)	13 (3.8)	76 (22.1)
29-31	753	385 (51.1) / 368 (48.9)	182 (24.2)	1410 (570-2580)	30 (29-31)	30 83-105)	81 (10.8)	131 (17.4)	49 (6.5)	27 (3.6)	122 (16.2)
32-34	999	514 (51.5) / 485 (48.5)	334 (33.4)	1725 (780-3450)	32 (32-34)	20 (1-180)	61 (6.1)	84 (8.4)	48 (4.8)	27 (2.7)	144 (14.4)
≥35	62	31 (50.0) / 31 (50.0)	19 (30.6)	1600 (1040-330)	35 (35-38)	23 (4-290)	4 (6.5)	1 (1.6)	0	0	7 (11.3)
p		0.879	<0.001^a^	<0.001^a^	<0.001^a^	<0.001^a^	<0.001^a^	<0.001^a^	<0.001^a^	0.280	0.016^a^

*n* number of cases, *BW* birth weight, *g* gram, *med* median, *min* minimum, *max* maximum, *GA* gestational age, *w* week, *O2* oxygen, *d* day, *BPD* broncho pulmonary dysplasia, *IVH* intraventricular hemorrhage, *NEC* necrotizing enterocolitis, *PDA* patent ductus arteriosus, *p* Comparison of the data in the relevant column between the BW or GA groups. Chi-Square or Mann-Whitney U tests; ^a^ Statistically significant result

### ROP rates

The number of infants with any stage of ROP was 952 (43.5%). The rates of mild ROP, type 2 ROP and type 1 ROP in infants was 751/2186 (34.4%), 51/2186 (2.3%) and 150/2186 (6.9%), respectively. The rate of ROP requiring treatment was 175/2186 (8.0%). Retinal laser diode photocoagulation was applied to 141 (80.6%) and intravitreal anti-VEGF (bevacizumab) injection was applied to 34 (19.4%) of 175 infants with ROP who needed treatment. ELBW infants had the highest rates of ROP (81%), severe (type 1) ROP (20.5%), and ROP requiring treatment (23.9%) compared to other BW groups (*p* < 0.001). In addition, ELGA infants had the highest rates of ROP (92.9%), severe (type 1) ROP (57.1%), and ROP requiring treatment (64.3%) compared to other GA groups (*p* < 0.001). ROP rates in BW and GA groups are presented in Table 2.

**Table 2:** The rates of ROP according to birth weight and gestational age

	n	ROP n (%)	Plus disease n (%)	Type of ROP			ROP requiring treatment n (%)
				Mild ROP n (%)	Type 2 ROP n (%)	Type 1 ROP n (%)	
Total	2186	952 (43.5)	150 (6.9)	751 (34.4)	51 (2.3)	150 (6.9)	175 (8.0)
BW (g)							
≤1000	347	281 (81.1)	71 (20.5)	184 (53.0)	26 (7.5)	71 (20.5)	83 (23.9)
1001-1750	1274	556 (43.6)	59 (4.6)	477 (37.4)	20 (1.6)	59 (4.6)	71 (5.6)
>1750	565	115 (20.4)	20 (3.5)	90 (15.9)	5 (0.9)	20 (3.5)	21 (3.7)
p		<0.001^a^	<0.001^a^	<0.001^a^			0.002^a^
GA (w)							
≤25	28	26 (92.9)	16 (57.1)	6 (21.4)	4 (14.3)	16 (57.1)	18 (64.3)
26-28	344	290 (84.3)	68 (19.8)	204 (59.3)	18 (5.2)	68 (19.8)	72 (20.9)
29-31	753	375 (49.8)	49 (6.5)	316 (42.0)	10 (1.3)	49 (6.5)	63 (8.4)
32-34	999	256 (25.6)	17 (1.7)	223 (22.3)	16 (1.6)	17 (1.7)	21 (2.1)
≥35	62	5 (8.1)	0 (0)	2 (3.2)	3 (4.8)	0 (0)	1 (1.6)
p		<0.001^a^	<0.001^a^	<0.001^a^			0.001^a^

*ROP* Retinopathy of prematurity, *n* number of cases, *BW* birth weight, *g* gram, *GA* gestational age, *w* week, *p* Comparison of the data in the relevant column between the BW or GA groups. Chi-Square test; ^a^ Statistically significant result

### Risk factors

The incidence of ROP increased statistically as BW (*p* < 0.001) and GA (*p* < 0.001) decreased (Table 2). The incidence of ROP requiring treatment also increased statistically with decrease of BW (*p* = 0.002) and GA (*p* = 0.001) (Table 2). The median BW (1300 vs 1600 g; *p* < 0.001) and GA (30 vs 32 w; *p* < 0.001) were statistically lower in infants with ROP compared to those without ROP (Table 3).

**Table 3:** Risk factors in infants with or without ROP

Risk factor	No ROP (*n* = 1234)	ROP (*n* = 952)	p
Gender: Male / Female, n (%)	640 (51.9) / 594 (48.1)	471 (49.5) / 481 (50.5)	0.268
Multiple gestations	368 (29.8)	247 (25.9)	0.046^a^
Twins / Triplets, n (%)	324 (26.3) / 44 (3.6)	205 (21.5) / 42 (4.4)	0.029^a^
BW (g), med (min-max)	1650 (680-3450)	1300 (540-2500)	<0.001^a^
GA (w), med (min-max)	32 (24-38)	30 (22-36)	<0.001^a^
Duration of O2 therapy (d), med (min-max)	20 (1-180)	39 (2-290)	<0.001^a^
Sepsis, n (%)	89 (7.2)	120 (12.6)	<0.001^a^
BPD, n (%)	132 (10.7)	181 (19.0)	<0.001^a^
IVH (≥Grade II), n (%)	64 (5.2)	89 (9.3)	<0.001^a^
NEC, n (%)	38 (3.1)	31 (3.3)	0.613
PDA, n (%)	199 (16.1)	162 (17.0)	0.333

*ROP* Retinopathy of prematurity, *n* number of cases, *BW* birth weight, *g* gram, *med* median, *min* minimum, *max* maximum, *GA* gestational age, *w* week, *O2* oxygen, *BPD* broncho pulmonary dysplasia, *IVH* intraventricular hemorrhage, *NEC* necrotizing enterocolitis, *PDA* patent ductus arteriosus, *p* Chi-Square or Mann-Whitney U tests; ^a^ Statistically significant result

The median duration of oxygen therapy increased statistically as BW (*p* < 0.001) and GA (*p* < 0.001) decreased (Table 1). The median duration of oxygen therapy (39 vs 20 days; *p* < 0.001) was longer in infants with ROP compared to those without ROP (Table 3).

The incidence of sepsis, BPD, and IVH (≥Grade II) increased statistically as BW (*p* < 0.001) and GA (*p* < 0.001) decreased (Table 1). The rates of sepsis, BPD, and IVH were statistically higher in infants with ROP compared to those without ROP (*p* < 0.001) (Table 3). While the NEC rate was higher in ELBW infants (*p* = 0.028), it did not differ according to GA and presence of ROP (Tables 1 and 3). While the rate of PDA was higher in both ELBW (*p* = 0.027) and ELGA (*p* = 0.016) infants, no difference was observed according to the presence of ROP (Tables 1 and 3).

### Multivariate logistic regression analysis

Compared to BW of > 1750 g, BW of ≤1000 g [OR (95% CI): 2.8 (1.8–4.4), *p* < 0.001] and BW of 1001–1750 g [1.6 (1.3–2.2), *p* < 0.001] were significant risk factors for ROP. Compared to GA of ≥35 w, GA of ≤25 w [66.5 (10.2–435.3), *p* < 0.001], GA of 26–28 w [34.7 (11.6–103.9), *p* < 0.001], GA of 29–31 w [10.5 (3.7–29.9), *p* < 0.001], and GA of 32–34 w [6.4 (2.3–18.4), *p* < 0.001] were significant risk factors for ROP. Additionally, increase in duration of oxygen therapy [1.03 (1.03–1.04), *p* < 0.001], presence of PDA [1.7 (1.2–2.4), *p* = 0.006], and presence of NEC [2.3 (1.2–4.1), *p* = 0.010] were determined as important risk factors for ROP.

## Discussion

Premature, especially ELBW and ELGA infants are known to have increased systemic morbidities, including ROP [2, 20, 21]. This study revealed that the rate of any stage ROP was 81.1% and the rate of ROP requiring treatment was 23.9% in ELBW infants. In the Early Treatment for Retinopathy of Prematurity (ETROP) study, the incidences of ROP were reported as 92.7 and 75.8%, respectively, in infants with a BW < 750 g and 750–1000 g. In addition, the incidences of prethreshold or worse ROP were reported as 51.9 and 31.7% in infants with a BW < 750 g and 750–1000 g, respectively [20]. While the rate of any stage ROP in ELBW infants was higher than in our previous report (81% vs 70.7%), the rate of severe ROP requiring treatment decreased (23.9% vs 30.2%) [13]. In another previous report, in which we compared two consecutive 5-year periods, we found that although the incidence of any stage ROP increased, the incidence of ROP requiring treatment decreased [22]. This contrast situation may be explained by the improvement in neonatal care practices despite the fact that younger babies are now being kept alive. In the TR-ROP study, which is a multicenter study conducted in our country, the rate of any stage ROP (68%) in ELBW infants was lower than in the current study, while the rate of ROP requiring treatment (20.8%) was similar [4]. A recent meta-analysis reported that the rates of any stage ROP, severe ROP and ROP requiring treatment in infants with a BW < 1000 g were 49, 24 and 18%, respectively [21]. The rates reported by this meta-analysis, which included 192 studies, are lower than those reported in our country [21]. The incidence of ROP in ELBW infants from different countries is given in Table 4. In the current study, in infants with a GA of ≤25 w, the rates of any stage ROP and ROP requiring treatment were 100, and 66.7%, respectively. Although there are not many studies on the incidence of ROP in ELGA infants, high rates have been reported from different countries, generally similar to the results of this study (Table 4). We think that the number of the infants with a GA of ≤25 w was small in the current study and that further studies are needed with a larger number of ELGA infants.

**Table 4:** The incidence of ROP in ELBW and ELGA infants from different countries

Author	Country	Year	BW or GA	Sample size	The rate of ROP	The rate of severe/treated ROP
Bas et al. 2018 [4]	Turkey	1	≤1000 g	1109	%68	%26
Alizadeh et al. 2015 [8]	Iran	5	≤1000 g	25	%47.1	-
Li et al. 2016 [9]	Taiwan	10	≤1000 g	167	%70.6	-
			≤25 w	56	%92.2	-
Yau et al. 2015 [10]	China	6	≤1000 g	131	%53.4	%14.5
Fortes Filho et al. 2013 [12]	Brazil	11	<1000 g	157	%37	%12.7
Demir et al. 2013 [13]	Turkey	9	<1000 g	225	%70.7	%30.2
Celebi et al. 2014 [14]	Turkey	3	≤1000 g	235	%75.5	%38.7
Bortea et al. 2021 [23]	Romania	3	<1000 g	62	%93.5	-
			≤25 w	9	%100	-
Zarei et al. 2019 [24]	Iran	1	<1000 g	160	%77.5	%66.2
Wu et al. 2018 [25]	China	3	<1000 g	60	%35	-
Fortes Filho et al. 2009 [26]	Brazil	4	≤1000 g	88	%48.9	%17
Li et al. 2016 [27]	China	3	≤1000 g	70	%45.7	%21.4
Isaza et al. 2012 [28]	Canada	4	≤1000 g	169	%73.3	%14.8
			≤25 w	86	%88.3	%24.4
Kong et al. 2012 [29]	Korea	7	<25 w	121	%98.4	%64.5
Gunn et al. 2012 [30]	Australia	18	≤25 w	373	%81.5	%15.1
Aikawa and Noro 2013 [31]	Japan	2	≤25 w	23	%87	%21.7
Current Study	Turkey	9	≤1000 g	347	%81.1	%23.9
			≤25 w	28	%92.7	%64.3

*ROP* Retinopathy of prematurity, *BW* birth weight, *GA* gestational age, *g* gram, *w* week

Lower GA and BW are major risk factors for the development of ROP. In the current study, it was found that lower GA and BW were independent risk factors for the development of ROP, and any stage ROP and severe ROP requiring treatment mostly developed in ELBW and ELGA infants. Logistic regression analysis confirmed that reductions in both GA and BW, more pronounced in GA, are important risk factors for the development of ROP. Studies from different countries have been published reporting that both lower GA and BW are significant risk factors for ROP with multivariate logistic regression analysis [4, 6, 8]. In addition, Li et al. and Bortea et al. determined only low BW as a risk factor for ROP [9, 23]. Yau et al. and Fortes Filho et al. reported that small GA was identified as a risk factor for ROP in ELBW infants [10, 12]. Zarei et al. also reported that more advanced stages of ROP were observed in extreme prematurity and ELBW infants, and GA was a risk factor for ROP in ELBW infants on multivariate analysis [24].

Increased duration of oxygen therapy was another risk factor for ROP. It has been reported in previous studies that supplementary oxygen administration and total days of oxygen are independent risk factors for ROP [4, 6, 8, 10]. Increased oxygen duration is a risk factor for ROP also confirmed by multivariate regression analysis in the current study. After birth, the encounter of the partially vascularized retina with hyperoxia leads to cessation of angiogenesis. Later, pathological neovascularization develops in the retina, which remains relatively hypoxic due to delayed vascularization relative to neuronal maturation [32]. Hyperoxia is a risk factor for eye, lung and cerebral injury in premature infants. In infants with GA of < 28 w, low saturation targets were associated with more deaths and more NEC, higher saturation targets (91–95%) were associated with more ROP. Despite this, it is recommended to target SpO2 at between 90 and 95% to reduce mortality in these infants [11, 33]. Stoll et al. reported that the rates of ROP decreased in infants with GA of 25–28 w, and this decrease might be related to oxygen saturation targets or to improved adherence to oxygen targets [1].

Some comorbidities may be risk factors for ROP. BPD is a lung disease that is defined as supplemental oxygen dependence beyond 28 days postpartum and results in prolonged oxygen exposure and is therefore associated with ROP. In our study BPD was found to be higher as BW and GA decreased, as well as in infants with ROP. Similar to some studies in the literature, higher rate of BPD was found in infants with ROP in our study [4, 6, 25, 34]. Yau et al. reported that BPD was identified as a risk factor for ROP in ELBW infants [10]. IVH was found to be higher in both ELBW and ELGA infants and infants with ROP. Although the association of IVH with ROP and severe ROP has been reported, the reason for the association is not known exactly [35]. In the current study, PDA was the most prevalent in ELBW and ELGA infants. While it could not be detected in the univariate analysis, the presence of PDA was found to be a risk factor for the development of ROP in the multivariate analysis. Several studies have reported that PDA or ligation of PDA is a risk factor for ROP [4, 6, 9, 25, 36]. Retinal hypoxia as a result of reduced perfusion in infants with PDA may lead to the development or progression of ROP. Weis et al. reported that while mortality was lower there was no difference in terms of ROP in babies with PDA who underwent ligation [37]. The frequency of NEC increased only with decreasing BW and was identified as a risk factor for the development of ROP by multivariate regression analysis. Li et al. and Kumar et al. reported an association between NEC and ROP by univariate regression analysis, but this association could not be demonstrated by multivariate regression analysis [9, 38]. Isaza et al. also reported that NEC is an independent risk factor for ROP [5]. Neonatal sepsis is one of the risk factors most commonly associated with the development of ROP [35]. Sepsis has also been identified as a risk factor for ROP in very low BW and ELBW infants [14, 25, 36]. Sepsis was found to be higher in both ELBW and ELGA infants and infants with ROP in the current study. Although its role in the pathogenesis is not fully elucidated, neonatal infection and perinatal inflammation are thought to play a role in the development of ROP [39].

## Conclusions

Currently, ELBW and ELGA infants with increasing chances of survival have a high rate of developing ROP and severe ROP. In these infants, prolonged duration of supplemental oxygen therapy and increased systemic comorbidities increase the risk of ROP. Consequently, the lower the BW and GA, and the longer the exposure to supplemental oxygen, the greater the risk of developing any ROP and severe ROP. The presence of concomitant neonatal sepsis, BPD, IVH (≥Grade II), PDA, and NEC further increases the risk of ROP. Infants with these risk factors should be followed very closely for the development of ROP, and their families should be informed about risks.

## Data Availability

All data generated or analysed during this study are included in this published article.

## Abbreviations

ROP: Retinopathy of prematurity
BW: Birth weight
GA: Gestational age
IVH: Intraventricular hemorrhage
PDA: Patent ductus arteriosus
RDS: Respiratory distress syndrome
BPD: Broncho pulmonary dysplasia
NEC: Necrotizing enterocolitis
ELBW: Extremely low birth weight
ELGA: Extremely low gestational age
g: Grams
w: Weeks
